# Latent Profile Analysis of Childhood Maltreatment and Neural Markers in Depression

**DOI:** 10.1001/jamanetworkopen.2025.25147

**Published:** 2025-08-04

**Authors:** Jessica Rowe, Nikita Nogovitsyn, Raegan Mazurka, Scott D. Squires, Stefanie Hassel, Jordan Poppenk, Katharine Dunlop, Mojdeh Zamyadi, Roumen V. Milev, Jane A. Foster, Stephen R. Arnott, Raymond W. Lam, Rudolf Uher, Susan Rotzinger, Sidney H. Kennedy, Benicio N. Frey, Kate L. Harkness

**Affiliations:** 1 Department of Psychology, Queen’s University, Kingston, Ontario, Canada; 2 Mood Disorders Treatment and Research Centre, St Joseph’s Healthcare, Hamilton, Ontario, Canada; 3 Centre for Depression and Suicide Studies, St Michael’s Hospital, Toronto, Ontario, Canada; 4 Department of Psychiatry, Dalhousie University, Halifax, Nova Scotia, Canada; 5 Centre for Neuroscience Studies, Queen’s University, Kingston, Ontario, Canada; 6 Department of Psychiatry, University of Calgary, Calgary, Alberta, Canada; 7 Hotchkiss Brain Institute, University of Calgary, Calgary, Alberta, Canada; 8 Mathison Centre for Mental Health Research and Education, University of Calgary, Calgary, Alberta, Canada; 9 School of Computing, Queen’s University, Kingston, Ontario, Canada; 10 Keenan Research Centre for Biomedical Science, St Michael’s Hospital, Toronto, Ontario, Canada; 11 Department of Psychiatry, University of Toronto, Toronto, Ontario, Canada; 12 Centre for Addiction and Mental Health, Toronto, Ontario, Canada; 13 Department of Psychiatry, Queen’s University, Providence Care Hospital, Kingston, Ontario, Canada; 14 Center for Depression Research and Clinical Care, Department of Psychiatry, University of Texas Southwestern Medical Center, Dallas; 15 Rotman Research Institute, Baycrest, Toronto, Ontario, Canada; 16 Indoc Research, Toronto, Ontario, Canada; 17 Department of Psychiatry, University of British Columbia, Vancouver, British Columbia, Canada; 18 Department of Psychiatry and Behavioural Neurosciences, McMaster University, Hamilton, Ontario, Canada

## Abstract

**Importance:**

The limited success of major depressive disorder (MDD) treatments is largely due to the disorder’s etiological and pathophysiological heterogeneity. Addressing this heterogeneity is essential for developing accurate prognostic models and personalized treatment strategies.

**Objective:**

To characterize MDD heterogeneity using a mechanism-first latent profile analysis based on environmental, neurostructural, and neurofunctional indicators, and to validate profiles via associations with MDD course, severity, and antidepressant treatment remission.

**Design, Setting, and Participants:**

This cross-sectional study used data from 2 Canadian Biomarker Integration Network in Depression (CAN-BIND) studies: CAN-BIND-1 (2014–2017), a multicenter outpatient antidepressant trial, and CAN-BIND-4 (2015–2018), a single-site study. Data analyses were completed from February to September 2024. Participants meeting *Diagnostic and Statistical Manual of Mental Disorders* (*Fourth Edition*) diagnostic criteria for unipolar depression were included. Individuals with lifetime bipolar, psychotic, substance use disorder, acute suicidality, and neurological disorders were excluded.

**Exposure:**

In CAN-BIND-1, patients received 10 to 20 mg of escitalopram daily; nonresponders at 8 weeks received aripiprazole augmentation for 8 additional weeks. CAN-BIND-4 was observational.

**Main Outcomes and Measures:**

Primary outcomes were latent profiles derived from childhood maltreatment (CM; semistructured interview); hippocampal, amygdala, thalamus structural volume (SV); anterior cingulate thickness (image segmentation); and DMN functional connectivity (average time series of the blood oxygen level–dependent signal). Secondary outcomes included associations with MDD course, symptom severity (including anhedonia, measured using Montgomery-Åsberg Depression Rating Scale), and remission rates.

**Results:**

In a sample of 309 adults with clinical depression (mean [SD] age, 33.81 [13.17] years; 206 female [66.67%]), 4 profiles emerged: (1) low CM and high SV, (2) low CM and low SV, (3) high CM and high SV, and (4) high CM and low SV with default mode network hypoconnectivity. Profile 4 was associated with the worst course, with the highest morbidity (mean number of years of morbidity, 19.91 years; 95% CI, 12.45-20.69 years), anhedonia (mean, 10.72; 95% CI, 9.74-11.70), and lowest remission rate (mean, 21.5%; 95% CI, 17.6%-23.5%) at week 16. Profile 3 had the highest remission rates (mean, 90.9%; 95% CI, 63.4%-118.0%).

**Conclusions and Relevance:**

In this cross-sectional study of 309 adults with depression, 4 latent profiles were identified. Default mode network hypoconnectivity defined profile 4, supporting its role as a key neural indicator of antidepressant response. CM was associated with both the highest and lowest remission rates, indicating it does not uniformly project negative outcomes and suggesting that neurobiological resilience in the context of childhood trauma may have contributed to more favorable clinical outcomes; further research is needed to refine clinical applications.

## Key Points

**Question:**

Are latent profiles of childhood maltreatment and neural markers of major depressive disorder (MDD) associated with clinical outcomes, including course, symptom severity, and remission, in antidepressant treatment?

**Findings:**

In this cross-sectional study of 309 individuals with depression, 4 profiles emerged: (1) low maltreatment and high neural volume, (2) low maltreatment and low neural volume, (3) high maltreatment and high neural volume, and (4) high maltreatment and low neural volume with default mode network hypoconnectivity. Profile 4 had the highest years of morbidity and lowest remission by week 16, while profile 3 showed the highest remission rates.

**Meaning:**

These findings underscore the value of identifying profiles of heterogeneity in MDD to inform personalized intervention.

## Teaser

This cross-sectional study uses latent profile analysis to characterize neurological and environmental heterogeneity in major depressive disorder (MDD) and identify associations of profiles with MDD course, severity, and antidepressant treatment remission.

## Introduction

Major depressive disorder (MDD) is the leading cause of disability worldwide, impacting an estimated 280 million individuals and incurring costs approaching $1 trillion annually.^1^ This substantial burden stems, in part, from inadequate treatment efficacy. Remission rates in antidepressant medication (ADM) trials are low, with approximately 50% of patients requiring further treatment beyond initial therapies and approximately 30% failing to achieve remission after 4 ADM trials.^2,3,4^ One key reason for this limited treatment success is the marked etiological and pathophysiological heterogeneity of MDD.^5,6,7^ Thus, adopting analytical methods that directly account for this heterogeneity is crucial for developing more effective prognostic models and personalized therapeutic approaches.

Latent profile analysis (LPA) is well-suited to capture heterogeneity in clinical populations by identifying subgroups with shared characteristics based on selected variables.^8^ Studies using LPA in depression have primarily focused on identifying symptom-based subgroups (eg, melancholic and atypical depression).^9^ These efforts have demonstrated limited predictive power for treatment outcomes,^5^ likely due to the principle of equifinality, whereby similar symptoms emerge from disparate pathophysiological mechanisms.^10^ As such, there has been a call to adopt a mechanism-first approach, which utilizes underlying risk markers as indicators,^5,11^ yet most studies adopting this approach have not validated the resulting subtypes against clinical outcomes, thereby limiting their prognostic relevance.^5^ Person-centered studies that have examined clinical outcomes, to date, have focused solely on neurofunctional indicators,^11,12,13^ limiting our understanding of MDD and the heterogeneity of MDD across multiple levels of analysis. Integrating across environmental, neuroanatomical, and neurofunctional indicators acknowledges the multidetermined nature of MDD and, in particular, the complex association between brain and environment.^14^

The current study has 2 primary aims. First, we used LPA to identify MDD subgroups based on empirically supported environmental and neural indicators. These indicators include severity of childhood emotional abuse, neglect, physical abuse, and sexual abuse^15^; left and right hippocampal,^16,17,18,19^ amygdala,^16,20^ and thalamus volume^21,22^; left and right rostral anterior cingulate cortex thickness^23,24^; and resting-state functional connectivity (rs-FC) of the default mode network (DMN).^25^ Second, we validated these profiles by examining their differential associations with MDD course, severity of symptom domains, and remission in an ADM treatment trial. LPAs are an iterative and generally exploratory process, so we had no specific hypotheses regarding the number or exact structure of the profiles. For our second aim, we hypothesized that profiles characterized by higher severity of childhood maltreatment (CM), lower brain volume and thickness, and hypoconnectivity in the DMN would be associated with an earlier age at first onset and greater years of morbidity, number of lifetime episodes, comorbidity, MDD symptom severity, and lower likelihood of remission following ADM treatment.

## Methods

### Participants

This cross-sectional study followed the Strengthening the Reporting of Observational Studies in Epidemiology (STROBE) reporting guideline. The final sample included participants ranging in age from 18 to 60 years drawn from 2 larger studies from the Canadian Biomarker Integration Network in Depression (CAN-BIND)^26,27,28^: CAN-BIND-1, a multisite clinical trial conducted between 2014 and 2017, and CAN-BIND-4, a naturalistic follow-up study conducted between 2015 and 2018 at one of the original sites. The original CAN-BIND studies received ethics approval from the relevant institutional review boards in line with national standards and the Declaration of Helsinki,^29^ and all participants provided informed consent for future research use of their data. The current study is therefore considered a secondary analysis of fully deidentified data and did not require additional ethics approval. All participants met *Diagnostic and Statistical Manual of Mental Disorders* (*Fourth Edition; DSM-IV*) diagnostic criteria for a unipolar depressive disorder.

**Figure 1.**
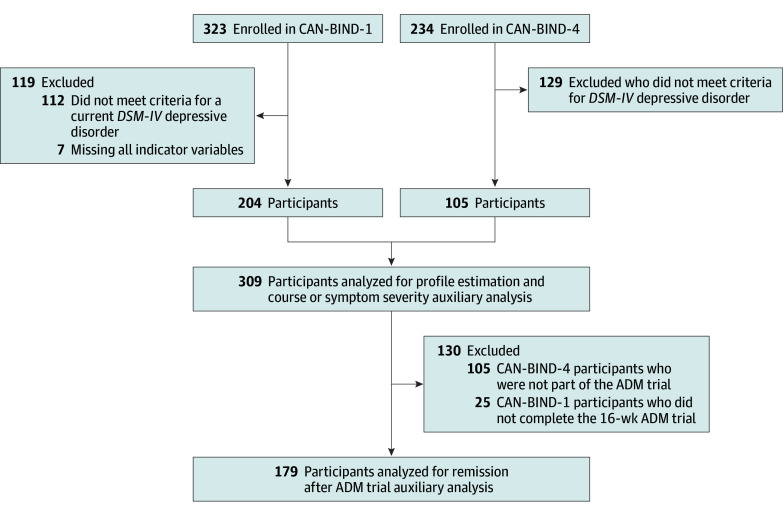
Participant Flow Diagram ADM indicates antidepressant medication; CAN-BIND, Canadian Biomarker Integration Network in Depression; *DSM-IV*, *Diagnostic and Statistical Manual of Mental Disorders, Fourth Edition*.

Full eligibility criteria for both CAN-BIND-1 and CAN-BIND-4, including detailed inclusion and exclusion protocols, have been published previously (Figure 1).^28,30^ In brief, CAN-BIND inclusion criteria for participants with depression were (1) episode duration of 3 or more months, (2) psychotropic-free for more than 5 half-lives, and (3) Montgomery-Åsberg Depression Rating Scale (MADRS) score 24 or greater. Exclusion criteria were (1) bipolar, psychotic, or substance use disorder history; (2) acute suicidality; (3) neurological issues, head trauma, or unstable health; and (4) pregnancy or breastfeeding. CAN-BIND-1 exclusion criteria also included (5) more than 4 nonresponses to treatments, (6) escitalopram (ESC) or aripiprazole (ARI) failure or intolerance, and (7) recent psychotherapy.

### Procedure

CAN-BIND-1 was a 6-site, open-label clinical trial. Patients began with 10 mg daily ESC, increasing to 20 mg daily at week 2 or 4 based on clinician judgment. Response at week 8 was defined as a 50% or greater MADRS reduction from baseline. Responders continued the effective ESC dose for an additional 8 weeks, while nonresponders received ARI augmentation (2-10 mg daily) for 8 weeks. CAN-BIND-4 was a 6-month observational study, with no standardized treatment protocol. Baseline data from both studies were used for profile estimation.

### Measures

#### Depression Diagnosis and Symptoms

The Mini International Neuropsychiatric Interview^31^ (CAN-BIND-1) or the Structured Clinical Interview for *DSM-IV* Axis I Disorders^32^ (CAN-BIND-4) was administered to determine current and past psychiatric diagnoses and course features. The MADRS^33^ was administered to assess depression symptom severity. We examined the association of our latent profiles with the following MADRS symptom domains: negative thoughts (ie, pessimistic thinking or suicidal ideation; α = .74), neurovegetative (ie, sleep problems, appetite, or inner tension; α = .93), detachment (ie, lack of interest, lassitude, or concentration; α = .78), and sadness (ie, reported and observed sadness; α = .94).^34^

Remission status at weeks 8 and 16 was defined as a MADRS score less than 10. Remission was used as the primary clinical outcome to ensure MDD symptoms fell below the threshold for a major depressive episode.^35^

#### CM

The Childhood Experiences of Care and Abuse Scale is a semistructured interview assessing history of emotional abuse (hostility and criticism), neglect (indifference to physical and/or emotional needs), physical abuse (violent acts), and sexual abuse (age-inappropriate or nonconsensual sexual activity) prior to age 18 years.^36^ Scales are subsequently rated by independent judges using a manual of rating rules and anchored exemplars. Emotional abuse and neglect were rated from 1 (little or none) to 4 (marked), and physical and sexual abuse were rated from 0 (none) to 4 (marked).

#### Neural Indicators

We have detailed the full CAN-BIND technical specifications, preprocessing, and quality control procedures for image acquisition, structural segmentation, and functional extraction in previous studies.^30^ Details are also provided in eAppendix 1 in the Supplement 1. Magnetic resonance imaging data were collected across 6 Canadian sites using standardized protocols, including aligned scanner settings, regular adherence checks, and a traveling human phantom to assess within- and between-site variance. Prior work using this dataset also directly examined volumetric differences across sites and found no significant differences in key structures (eg, hippocampus; *P* > .96).^37^

#### Structural Segmentation

Image segmentation was conducted using FreeSurfer version 7.1.1 (Laboratory for Computational Neuroimaging at the Athinoula A. Martinos Center for Biomedical Imaging). Probabilistic atlases estimated hippocampus, amygdala, and thalamus structural volume (SV), while cortical thickness measures were derived from the Desikan-Killiany atlas.^38^ Quality control was based on the Enhancing Neuroimaging Genetics Through Meta-Analysis consortium guidelines (eAppendix 1 in Supplement 1).^39^

#### rs-FC

The average time series of the blood oxygen level–dependent signal was extracted from regions of interest using a modified version of the Multiresolution Intrinsic Segmentation Template 122-region functional atlas,^40^ following preprocessing in the Optimization of Preprocessing Pipelines for Neuroimaging (see eAppendix 2 in Supplement 1 for full details). rs-FC analyses focused on the DMN, using a subset of 4 functional clusters, selected based on the atlas’s hierarchical parcellation, reflecting canonical DMN subdivisions.^41^ Pairwise rs-FC was calculated using Pearson correlation coefficient across region of interest time courses and Fisher-z transformed. Measurements ±2 SDs from the mean were excluded (2 measurements).

### Statistical Analysis

Data analysis was completed in February to September 2024. To test our first aim, an LPA was conducted using the 16 indicator variables in Mplus version 8.3 (Muthén & Muthén), employing full-information maximum likelihood estimation.^42^ All indicators were continuous and were *z*-transformed to facilitate interpretation. Coverage ranged from 0.71 to 0.92, exceeding the recommended minimum of 0.1, which allowed for the inclusion of participants with partial data.^43^ Model selection was based on fit indices, parsimony, and theoretical interpretability across covariance structures (eTable 1 and eAppendix 3 in Supplement 1). After selecting the optimal latent profile solution, Wald χ^2^ tests examined significant differences across profile indicators. A Bonferroni correction was applied to control for type I error across the 6 tests, resulting in an adjusted significance threshold of *P* < .0083.

To test our second aim, we used the Bolck-Croon-Hagenaars method for continuous outcomes and distal categorical for categorical outcomes to assess profile differences in MDD course, symptom severity, and ADM treatment remission.^44,45^ These methods retain latent probabilities, minimizing classification error compared with modal or 1-step approaches.^46^ Treatment remission analyses were limited to CAN-BIND-1 participants, while all participants were included in all other outcome analyses. A Bonferroni correction was again applied within each auxiliary model for the 6 comparisons (*P* < .0083).

## Results

### LPA

**Figure 2.**
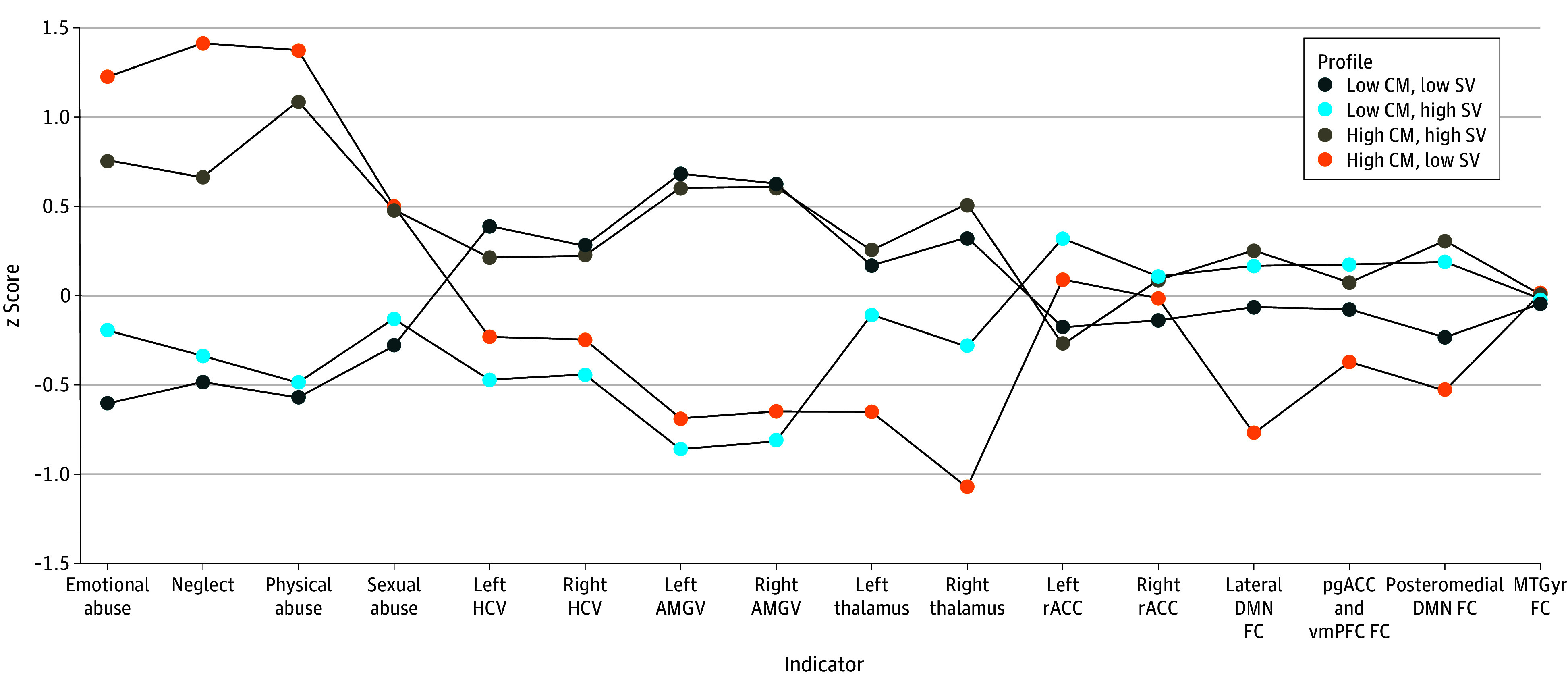
Profile Plot of the 4-Profile Solution of Childhood Maltreatment and Neural Markers of Major Depressive Disorder AMGV, amygdala volume; CM, childhood maltreatment; DMN, default mode network; FC, functional connectivity; HCV, hippocampal volume; MTGyr, medial temporal gyrus; pgACC, perigenual anterior cingulate cortex; rACC, rostral anterior cingulate cortex; SV, structural volume; THV, thalamus volume; vmPFC, ventromedial prefrontal cortex.

The final sample included 309 adult participants with depression (mean [SD] age, 33.81 [13.17] years; 206 female [66.67%]), including 204 from CAN-BIND-1 and 105 from CAN-BIND-4. Descriptive statistics are presented in Table 1. The 4-profile LPA, using a profile-invariant diagonal covariance structure (eAppendix 3 in Supplement 1), was selected as the optimal solution based on model comparison indices, satisfactory entropy, model parsimony, signs of possible data overfitting with increasing latent profiles, and interpretation of profiles (Table 2). As displayed in Figure 2, the 4 profiles were labeled as follows: (1) low CM and high SV (approximately 113 participants [36.57%]), (2) low CM and low SV (approximately participants [32.36%]), (3) high CM and high SV (approximately 58 participants [19.09%]), and (4) high CM and low SV (approximately 34 participants [11.07%]). Counts reflect modal assignment based on posterior probabilities.

**Table 1.** Indicator Differences Across Latent Profiles

Variable	Full sample, mean (SD)	Profile 1: low CM and high SV, mean (95% CI)	Profiles with significant differences^a^	Profile 2: low CM and low SV, mean (95% CI)	Profiles with significant differences^a^	Profile 3: high CM and high SV, mean (95% CI)	Profiles with significant differences^a^	Profile 4: high CM and low SV, mean (95% CI)	Profiles with significant differences^a^	Omnibus, χ^2^ test^b^	*P* value
Childhood maltreatment											
Emotional abuse	1.13 (1.30)	−0.60 (−0.78 to −0.46)	2, 3, and 4	−0.19 (−0.41 to 0.03)	1,3, and 4	0.76 (0.43 to 1.08)	1 and 2	1.22 (0.87 to 1.58)	1 and 2	106.62	<.001
Neglect	0.87 (1.41)	−0.48 (−0.65 to −0.32)	2 and 4	−0.34 (−0.53 to −0.14)	2 and 4	0.66 (0.21 to 1.11)	1 and 2	1.41 (1.03 to 1.80)	1 and 2	94.98	<.001
Physical abuse	2.17 (1.03)	−0.57 (−0.72 to −0.42)	2 and 4	−0.49 (−0.66 to −0.32)	2 and 4	1.09 (0.76 to 1.42)	1 and 2	1.38 (1.07 to 1.68)	1 and 2	198.60	<.001
Sexual abuse	1.74 (0.90)	−0.28 (−0.46 to −0.11)	3 and 4	−0.13 (−0.35 to 0.09)^a^	3	0.48 (0.11 to 0.86)	1 and 2	0.50 (−0.01 to 1.02)	1	22.51	<.001
SV^c^											
Left hippocampus	3.20 (1.27)	0.39 (−0.01 to 0.79)	2	−0.47 (−0.75 to −0.20)	1	0.21 (−0.09 to 0.52)	2	−0.23 (−0.63 to 0.16)	NA	14.85	.002
Right hippocampus	3.37 (1.12)	0.28 (−0.06 to 0.62)	2	−0.44 (−0.67 to −0.21)	1	0.22 (−0.02 to 0.47)	2	−0.25 (−0.59 to 0.10)^a^^,^^b^	NA	19.91	<.001
Left amygdala	1.50 (0.75)	0.68 (0.43 to 0.93)	2 and 4	−0.86 (−1.17 to −0.56)	1 and 3	0.60 (0.39 to 0.82)	2 and 4	−0.69 (−0.95 to −0.43)	1 and 3	265.75	<.001
Right amygdala	1.55 (0.77)	0.63 (0.41 to 0.85)	2 and 4	−0.82 (−1.14 to −0.49)	1 and 3	0.61 (0.37 to 0.85)	2 and 4	−0.65 (−0.96 to −0.33)	1 and 3	185.78	<.001
Left thalamus	6.51 (2.34)	0.17 (−0.06 to 0.40)	4	−0.11 (−0.34 to 0.13)^a^	NA	0.25 (−0.08 to 0.58)	4	−0.65 (−1.06 to −0.24)	1 and 3	16.31	.001
Right thalamus	6.59 (2.39)	0.32 (0.11 to 0.56)	2 and 4	−0.29 (−0.56 to −0.02)	1 and 4	0.51 (0.24 to 0.79)	2 and 4	−1.07 (−1.46 to −0.69)	1, 2, and 3	55.03	<.001
Cortical thickness											
Left rostral ACC	2.77 (0.19)	−0.18 (−0.40 to 0.05)	2	0.32 (0.06 to 0.58)	1 and 3	−0.27 (−0.64 to 0.09)	2	0.09 (−0.24 to 0.43)	NA	10.88	.01
Right rostral ACC	2.77 (0.20)	−0.14 (−0.38 to 0.10)	NA	0.11 (−0.12 to 0.33)	NA	0.09 (−0.35 to 0.53)	NA	−0.02 (−0.42 to 0.39)	NA	1.92	.59
Resting-state functional connectivity											
Lateral DMN	0.86 (0.37)	−0.06 (−0.39 to 0.26)	4	0.17 (−0.14 to 0.48)	4	0.25 (−0.06 to 0.56)	4	−0.77 (−1.17 to −0.38)	1, 2, and 3	17.34	<.001
Perigenual ACC and ventromedial PFC	1.02 (0.35)	−0.08 (−0.39 to 0.24)	NA	0.17 (−0.10 to 0.45)	NA	0.07 (−0.29 to 0.44)	NA	−0.37 (−0.87 to 0.12)	NA	3.28	.35
Posteromedial DMN	0.62 (0.29)	−0.23 (−0.43 to −0.05)	NA	0.19 (−0.08 to 0.46)	4	0.31 (−0.05 to 0.66)	4	−0.53 (−0.97 to −0.09)	2 and 3	11.38	.009
Middle temporal gyrus	0.49 (0.26)	−0.04 (−0.39 to 0.24)	NA	−0.02 (−0.33 to 0.30)	NA	0.01 (−0.35 to 0.36)	NA	0.01 (−0.45 to 0.47)	NA	0.09	.99

Abbreviations: ACC. anterior cingulate cortex; CM, childhood maltreatment; DMN, default mode network; NA, not applicable; PFC, ventromedial prefrontal cortex; SV, structural volume.

^a^ Significant pairwise differences between profiles based on Wald χ^2^ parameter constraint tests (*P* < .0083; Bonferroni-corrected).

^b^ Degrees of freedom = 48 for all measures.

^c^ SVs are presented in centimeters cubed prior to regressing on total brain volume for ease of interpretation.

**Table 2.** Latent Profile Analysis of Comparative Fit Indices and Model Characteristics

No. of profiles^a^	Akaike information criterion	BIC	Sample size–adjusted BIC	*P* value		Final log-likelihood	Entropy	Smallest profile (% of total sample)
				Vuong-Lo-Mendell-Rubin LRT	Parametric bootstrap LRT			
2	12 003.48	12 186.42	12 031.01	<.001	*<*.001	−5952.74	0.83	31.07
3	11 824.58	12 070.98	11 861.65	.16	*<*.001	−5846.28	0.79	26.54
4^b^	11 725.13	12 034.99	11 771.75	.04	<.001	−5779.56	0.82	11.97
5	11 648.83	12 022.16	11 705.00	.32	<.001	−5724.41	0.83	8.41
6	11 534.01	11 970.82	11 599.74	.70	<.001	−5650.01	0.84	6.15
7	11 565.06	11 965.24	11 540.36	.39	<.001	−5598.53	0.86	1.62

Abbreviations: BIC, bayesian information criterion; LRT, likelihood ratio test.

^a^ All profiles were estimated with profile invariant diagonal covariance structure.

^b^ Chosen as optimal profile solution.

### Differences in Indicators Across Profiles

Profile 1 (low CM and high SV) had significantly lower severity of emotional abuse (mean, −0.60; 95% CI, −0.78 to −0.46) than all other profiles and significantly lower neglect (mean, −0.48; 95% CI, −0.65 to −0.32), physical abuse (mean, −0.28; 95% CI, −0.46 to −0.11), and sexual abuse (mean, −0.28; 95% CI, −0.46 to −0.11) severity than the high CM profiles (Table 2 and Figure 2). Profile 1 had significantly higher amygdala (left: mean, 0.68; 95% CI, 0.43 to 0.93; right: mean, 0.63; 95% CI, 0.41 to 0.85), hippocampus (left: mean, 0.39; 95% CI, −0.01 to 0.79; right: mean, 0.28; 95% CI, −0.06 to 0.62), and thalamus (left: mean, 0.17; 95% CI, −0.06 to 0.40; right: mean, 0.32; 95% CI, 0.11 to 0.56) volumes and lower left rostral anterior cingulate cortex thickness (mean, −0.18; 95% CI, −0.40 to 0.05) than the low CM and low SV profile and significantly higher amygdala and thalamus volumes and lateral DMN connectivity (mean, −0.06 95% CI, −0.39 to 0.26) than the high CM and low SV profile.

Profile 2 (low CM and low SV) had significantly lower emotional abuse (mean, −0.19; 95% CI, −0.41 to 0.03), neglect (−0.34; 95% CI, −0.53 to −0.14), and physical abuse (mean, −0.49; 95% CI, −0.66 to −0.32) severity than the high CM profiles, and significantly lower hippocampal (left: mean, −0.47; 95% CI, −0.75 to −0.20; right: mean, −0.44; 95% CI, −0.67 to −0.21), amygdala (left: mean, −0.86; 95% CI, −1.17 to −0.56; right: −0.82; 95% CI, −1.14 to −0.49), and right thalamus (mean, −0.29; 95% CI, −0.56 to −0.02) volumes than the 2 high SV profiles. Additionally, profile 2 had significantly higher rs-FC in the lateral (mean, 0.17; 95% CI, −0.14 to 0.48) and posteromedial DMN (mean, 0.19; 95% CI, −0.08 to 0.46) than the high CM and low SV profile.

Profile 3 (high CM and high SV) had significantly higher severity of all maltreatment types (emotional abuse: mean, 0.76; 95% CI, 0.43 to 1.08; neglect: mean, 0.66; 95% CI, 0.21 to 1.11; physical abuse: mean, 1.09; 95% CI, 0.76 to 1.42; sexual abuse: mean, 0.48; 95% CI, 0.11 to 0.86) than the low CM profiles, significantly higher amygdala (left: mean, 0.60; 95% CI, 0.39 to 0.82; right: mean, 0.61; 95% CI, 0.37 to 0.85) and right thalamus volumes (mean, 0.51; 95% CI, 0.24 to 0.79) than the low SV profiles, and significantly higher hippocampal volume (left: mean, 0.21; 95% CI, −0.09 to 0.52; right: mean, 0.22; 95% CI, −0.02 to 0.47) than the low CM and low SV profile. Compared with the high CM and low SV profile, profile 3 had significantly higher left thalamus volume (mean, 0.25; 95% CI, −0.08 to 0.58), lower left rostral anterior cingulate cortex thickness (mean, −0.27; 95% CI, −0.64 to 0.09), and higher lateral (mean, 0.25; 95% CI, −0.06 to 0.56) and posteromedial (mean, 0.01; 95% CI, −0.35 to 0.36) DMN rs-FC connectivity.

Profile 4 (high CM and low SV) had significantly higher emotional abuse (mean, 1.22; 95% CI, 0.87 to 1.58), neglect (mean, 1.41; 95% CI, 1.03 to 1.80), and physical abuse (mean, 1.38; 95% CI, 1.07 to 1.68) severity than both low CM profiles and significantly higher sexual abuse severity (mean, 0.50; 95% CI, −0.01 to 1.02) than the low CM and high SV profile. This profile had significantly lower amygdala (left: mean, −0.69; 95% CI, −0.95 to −0.43; right: mean, −0.65; 95% CI, −0.96 to −0.33) and thalamus (left: mean, −0.65; 95% CI, −1.06 to −0.24; right: mean, −1.07; 95% CI, −1.46 to −0.69) volumes than both high SV profiles, significantly lower lateral DMN rs-FC connectivity (mean, −0.77; 95% CI, −1.17 to −0.38) than all other profiles, and significantly lower posteromedial rs-FC (mean, −0.53; 95% CI, −0.97 to −0.09) than the low CM and low SV and high CM and high SV profiles.

### Clinical Differences Across Profiles

Descriptive statistics for the clinical variables available in the full sample are presented in eTable 2 in Supplement 1. No significant profile differences emerged for age of onset, lifetime episodes, comorbidities, or severity of negative thoughts or neurovegetative symptoms (eTable 3 in Supplement 1). Profile 4 (high CM and low SV) had a significantly higher number of years of morbidity (mean, 19.91 years; 95% CI, 12.45-20.69 years) than the 2 low CM profiles and significantly higher severity of detachment symptoms (mean, 10.72; 95% CI, 9.74-11.70) than the low CM and high SV profile. Conversely, the low CM and high SV profile had significantly lower severity of sadness symptoms (mean, 5.98; 95% CI, 5.57-6.39) than all other profiles.

**Figure 3.**
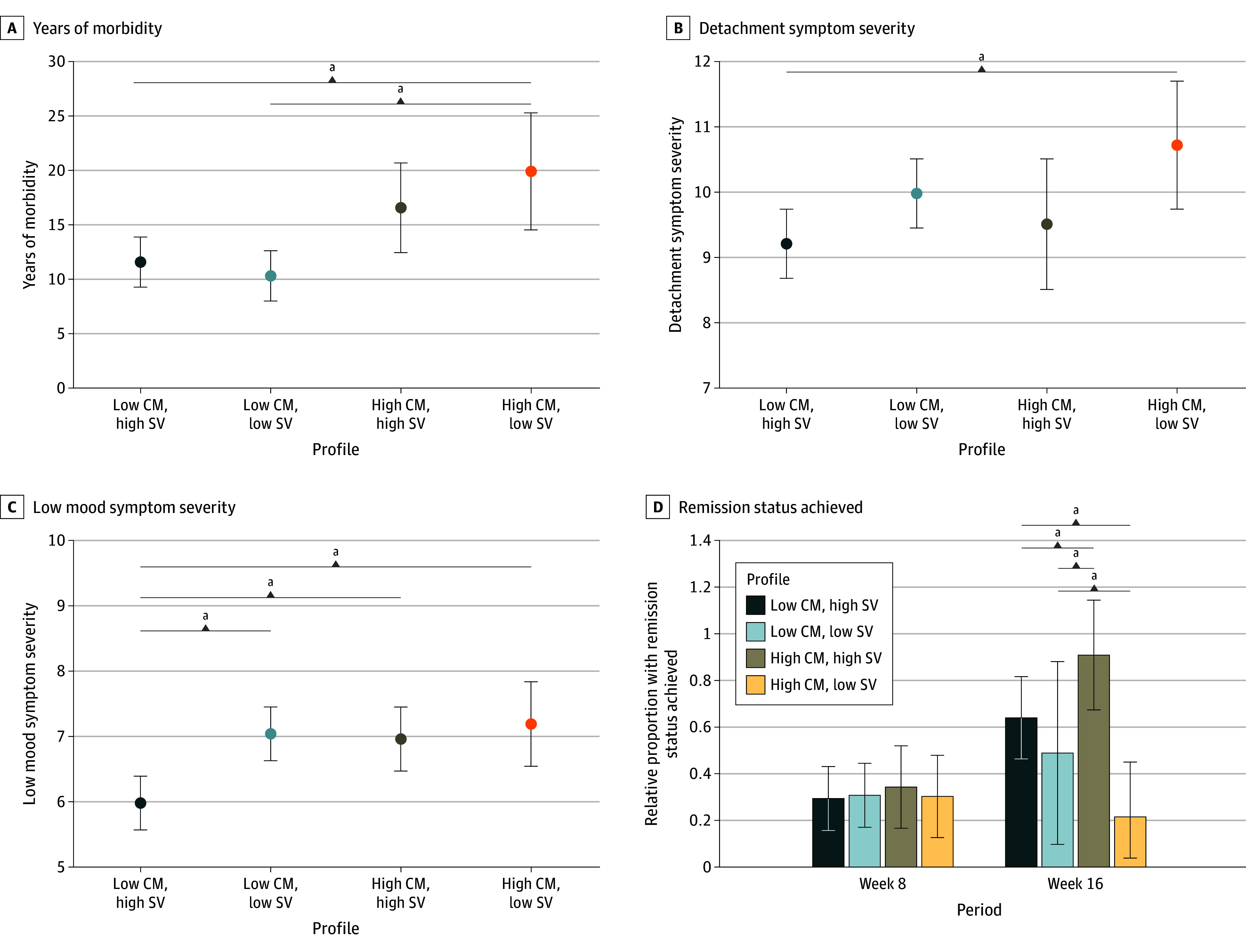
Clinical Outcome Differences Between Latent Profiles Data summarized as means with SD (A-C) or probability of profile membership with 95% CIs (D). CM indicates childhood maltreatment; SV, structural volume. ^a^For continuous variables, denotes the Bolck, Croon, and Hagenaars–weighted equality of means test (Wald χ^2^) between each profile and the profile number indicated at *P* < .0083. For categorical variables, denotes equality tests of probabilities (Wald χ^2^) tests between each profile and the profile number indicated at *P* < .0083.

Among the 179 CAN-BIND-1 participants, remission rates were 17.8% at week 8 (34 participants) and 31.7% (57 participants) at week 16. There were no significant differences in remission rates across profiles at week 8. However, significant differences emerged after ARI augmentation at week 16 (Figure 3D). The high CM and low SV profile (profile 4) was significantly more likely to contain nonremitters than high SV profiles, and had the lowest remission rate (mean, 21.5%; 95% CI, 17.6%-23.5%). In contrast, the high CM and high SV profile (profile 3) had the highest probability of remission (mean, 90.9%; 95% CI, 63.4%-118.0%) and was significantly more likely to contain remitters than all other profiles.

## Discussion

This multicenter cross-sectional study identified 4 distinct latent profiles of depressive disorders in a large sample of adults that differed significantly by CM history; hippocampal, amygdala, and thalamic volumes; and DMN connectivity. These profiles can be interpreted as reflecting a continuum of risk given their alignment with neurobiological and environmental risk factors for depression. The profile reflecting the lowest relative risk was characterized by low CM severity, high brain volumes, and DMN hyperconnectivity (profile 1). In contrast, the profile with the highest relative risk was characterized by high CM severity, low brain volumes, and DMN hypoconnectivity (profile 4). In the middle were a profile characterized by SV risk markers only (profile 2) and a profile characterized by environmental risk markers only (profile 3). Therefore, the current results suggest that the marked heterogeneity in MDD can be described as a dissociation between neural and environmental risk indicators.

Consistent with hypotheses, the highest risk profile (profile 4), characterized by severe CM, low brain volumes, and DMN hypoconnectivity, was associated with the worst clinical course. This profile had the greatest years of morbidity, the highest severity of detachment symptoms (ie, anhedonia), and lowest remission rates in a 16-week ESC trial, even after ARI augmentation. Importantly, this profile was associated with high environmental risk across all maltreatment types and low structural and resting-state functional integrity. In contrast, profiles with isolated environmental or neural risk did not differ significantly from the lowest risk profile in terms of years of morbidity or anhedonia, underscoring the importance of integrating multiple levels of analysis to identify prognostically meaningful MDD subgroups.^47^

The neural marker that characterized the highest risk profile was hypoconnectivity in the DMN. DMN hypoconnectivity is associated with impairments in self-referential processing and overgeneral autobiographical memory.^48,49,50^ Thus, our results are consistent with previous studies, which show that deficits in processing positive autobiographical information are associated with anhedonia in MDD,^51^ and that overgeneral autobiographical memory significantly predicts a more recurrent and persistent course of MDD.^52^ Furthermore, DMN hypoconnectivity was the key neural indicator that differentiated this highest risk profile from the other low SV profile, which, in contrast, had a low severity of CM (profile 2). Thus, our results are also consistent with studies suggesting that impairments in self-referential processing and overgeneral memory may mediate the pathway from maltreatment to later MDD.^53^

Further, DMN rs-FC has been identified as a candidate biomarker for ADM response across various clinical trial settings, medications, and analytic strategies.^54^ Specifically, more robust connectivity within the DMN has been associated with a better response to short-term ADM treatment, and to ESC and ARI in particular, whereas DMN hypoconnectivity has been associated with nonresponse to ADMs.^12,55^ For instance, Tozzi and colleagues^13^ identified an rs-FC subtype characterized by DMN hyperconnectivity, which also exhibited superior treatment outcomes. These parallels reinforce the robustness of our results and suggest emerging consensus across independent samples and methodologies regarding the prognostic significance of DMN connectivity patterns. Converging evidence also suggests that the therapeutic effect of ARI may operate through the down-modulation of DMN functional connectivity.^56,57^ While this modulation is potentially effective for those with hyperconnectivity in the DMN, it may be less beneficial, or even counterproductive, for those presenting with DMN hypoconnectivity, highlighting the need for personalized treatment approaches.

The highest risk profile (profile 4) was also the smallest in terms of estimated membership (11.07% of the sample). This finding challenges the prevailing view that CM causes alterations in brain volume and function and, thus, early environmental and neural indicators should track together.^58,59,60^ CM was not associated with low brain volume for an even greater number of individuals in our sample (19.09% in profile 3). However, it is possible that the neural sequelae examined here are specific to maltreatment experienced during particular developmental periods and/or to a greater frequency or chronicity of maltreatment.^61^ It is also possible that CM in profile 3 is associated with neuropathology in structures and networks not examined here.^58^ Therefore, future LPA studies providing more fine-grained characterization of maltreatment and a broader set of neural indicators are warranted.

However, profile 3 also had the highest likelihood of remission in the ADM trial, significantly higher even than the lowest risk profile (profile 1). This challenges the meta-analytic evidence identifying CM as a negative prognostic indicator^13,62^ and suggests an intriguing dissociation. Specifically, CM in the presence of neural markers indicating low SV and DMN hypoconnectivity was associated with the lowest remission rates. In direct contrast, CM in the absence of these neural indicators was associated with the highest likelihood of remission and thus suggests a potential protective effect of higher SVs. As such, an intriguing question for future research is whether neurobiological resilience in the face of childhood trauma is a potential mechanism underlying superior remission rates.^63,64,65^

### Limitations

Findings should be considered in light of the following limitations. First, the profiles identified here require validation across independent samples.^66^ Although the sample was relatively large for neuroimaging indicators,^67^ replication in a larger and more diverse cohort is needed, particularly to confirm the stability of the smallest profile (profile 4). Second, retrospective reports of CM may be biased by current symptoms. However, the contextual interview approach with independent ratings is the benchmark for retrospective assessment of CM.^30,68^ Third, prospective longitudinal studies are needed to examine the development and maintenance of these profiles, with large-scale longitudinal cohorts providing valuable opportunities for person-centered growth modeling.^69^ Fourth, the lowest risk profile (profile 1) had the largest membership (36.5%), indicating that key contributors to MDD cause and pathophysiology may not have been captured. Therefore, future research integrating an even larger set of indicators representing broader domains of functioning is warranted (eg, neurogenetic markers and information processing).

## Conclusions

In this cross-sectional study of 309 individuals with depression, we provided novel evidence for 4 latent profiles in MDD, differentiated by early environmental history, SV, and rs-FC in the DMN. The emergence of these profiles underscores the heterogeneity of MDD and its multidetermined nature. Profiles had prognostic value; DMN hypoconnectivity paired with CM was associated with greater illness severity and lower remission rates, while maltreatment without neural risk was associated with the highest likelihood of remission. These findings demonstrate the clinical utility of subgroup identification in MDD, elucidating its heterogeneous cause and informing more targeted, personalized interventions based on distinct neurobiological and environmental profiles.

## Notes

Supplement 1. **eAppendix 1.** Structural Image Acquisition and Parameters **eAppendix 2.** Resting-State Functional Connectivity Image Acquisition and Parameters **eTable 1.** LPA Comparative Fit Indices and Model Characteristics **eAppendix 3.** Optimal Profile Solution Selection **eTable 2.** Descriptive Raw Score Statistics for the Clinical Variables in the Full Sample **eTable 3.** Clinical Characteristics and Symptom Severity Differences Between Latent Profile Classes
eReferences.

Supplement 2.
Data Sharing Statement
