# Supplementary material for: Latent Profile Analysis of Childhood Maltreatment and Neural Markers in Depression
Source: JAMA Netw Open. 2025 Aug 4;8(8):e2525147. doi: 10.1001/jamanetworkopen.2025.25147 (PMC12322798; doi:10.1001/jamanetworkopen.2025.25147)
Supplement: Supplement 1. — eAppendix 1. Structural Image Acquisition and Parameters eAppendix 2. Resting-State Functional Connectivity Image Acquisition and Parameters eTable 1. LPA Comparative Fit Indices and Model Characteristics eAppendix 3. Optimal Profile Solution Selection eTable 2. Descriptive Raw Score Statistics for the Clinical Variables in the Full Sample eTable 3. Clinical Characteristics and Symptom Severity Differences Between Latent Profile Classes eReferences. [file jamanetwopen-e2525147-s001.pdf]

Supplemental Online Content

Rowe J, Nogovitsyn N, Mazurka R, et al. Latent profile analysis of childhood maltreatment and neural markers in depression. *JAMA Netw. Open*. 2025;8(8):e2525147. doi:10.1001/jamanetworkopen.2025.25147

eAppendix 1. Structural Image Acquisition and Parameters
eAppendix 2. Resting-State Functional Connectivity Image Acquisition and Parameters
eTable 1. LPA Comparative Fit Indices and Model Characteristics
eAppendix 3. Optimal Profile Solution Selection
eTable 2. Descriptive Raw Score Statistics for the Clinical Variables in the Full Sample
eTable 3. Clinical Characteristics and Symptom Severity Differences Between Latent Profile Classes
eReferences.

This supplemental material has been provided by the authors to give readers additional information about their work.

© 2025 Rowe J et al. *JAMA Network Open*.

eAppendix 1. Structural Image Acquisition and Parameters

Whole-brain T1-weighted structural scans were performed. The parameters involved TR =6.4-1900 ER ms; =2.2-3.4 ms; FA=8-15°; TI=450-950 ms; FOV=256 mm; matrix 220 x 220 and 256 x 256; 155-192 slices recorded at 1 mm3 (3:30-9:53 min).

A total of 250 neuroimaging-derived metrics (e.g., regional volumes and cortical thickness) were compiled and subjected to a quality control procedure based on ENIGMA consortium guidelines.[1] Outliers were identified based on deviations (±2 SD) from the sample mean for total gray matter, intracranial volume, and their ratio. Flagged cases were visually inspected in Freeview and compared to a standard anatomical atlas; any variables with inaccurate structural labelling were excluded. To adjust for total brain volume (TBV), volumetric measures were regressed on TBV, and standardized residuals were used in analyses.[2]

© 2025 Rowe J et al. *JAMA Network Open*.

eAppendix 2. Resting-State Functional Connectivity Image Acquisition and Parameters

Whole-brain T1-weighted rs-FC scans were also performed. The parameters involved TR = 2000.0 ms, ER = 30.0 ms, FA = 75°, FOV = 256-1536, matrix 64 x 64, 34-36 slices recorded at 4mm3 (10:00 min).

The OPPNI preprocessing workflow[3,4] included 14 primary computational steps, as follows:

1. DICOM images were converted to NIfTI format using the MRIcron converter (dcm2nii);
2. To identify the BOLD volume with the least head displacement, principal component analysis (PCA) was performed, where principal components were multiplied by corresponding eigenvalues. The data was then centered based on the median factor derived from all factors across each time-point. For each volume, the mean distance from the center was calculated, and the volume with the smallest distance was designated as the reference volume for minimal head displacement, used in the subsequent motion correction step (details in technical report)[5];
3. Rigid-body motion correction (MOTCOR) was applied using the AFNI command 3dvolreg, aligning all volumes to the reference volume identified in step 2, utilizing a weighted least-squares cost function and Fourier interpolation;
4. Censoring (CENSOR) was conducted using an outlier identification algorithm4 applied to all fMRI volumes. This algorithm, which uses a sliding time-window, interpolates data to replace outlier volumes based on neighboring volumes (available online at http://nitrc.org/projects/spikecor_fmri);
5. Slice-timing correction (TIMECOR) was completed using AFNI's 3dTshift module;
6. Spatial smoothing across two MRI scans was standardized with the AFNI module 3dBlurToFWHM (FWHM=6mm, in three spatial directions);
7. Binary masks were generated using AFNI's 3dAutomask and applied to all EPI volumes;
8. Neuronal tissue masking was conducted with the initial component of the PHYCAA+ algorithm5 (available at http://nitrc.org/projects/phycaa_plus);
9. Low-frequency temporal trends were modeled using a Legendre polynomial basis;
10. Head motion effects were calculated using subject motion parameter estimates (MPEs) from the MOTCOR process (step 3);
11. Global signal regression (GSPC1) was performed by removing the first principal component from the fMRI data;
12. Physiological noise was removed in a data-driven manner using the second part of the PHYCAA+ algorithm;
13. Low-pass filtering (LOWPASS) was applied with a linear filter to eliminate BOLD frequencies above 0.10Hz;
14. Finally, spatial normalization to the MNI152 template (sNORM) was achieved using the FSL FLIRT module to facilitate the combination of results across subjects

© 2025 Rowe J et al. *JAMA Network Open*.

eTable 1. LPA Comparative Fit Indices and Model Characteristics

No. of profiles	AIC	BIC	sBIC	VLMR-LRT	PBLRT	Finallog-likelihood	Entropy	Smallest profile (% of total sample)
Profile Invariant Diagonal								
2	12003.48	12186.42	12031.01	$p < .001$	$p < .0001$	-5952.74	.83	31.07
3	11824.58	12070.98	11861.65	$p = .16$	$p < .0001$	-5846.28	.79	26.54
4[a]	11725.13	12034.99	11771.75	$p = .04$	$p < .0001$	-5779.56	.82	11.97
5	11648.83	12022.16	11705.00	$p = .32$	$p < .0001$	-5724.41	.83	8.41
6	11534.01	11970.82	11599.74	$p = .70$	$p < .0001$	-5650.01	.84	6.15
7	11565.06	11965.24	11540.36	$p = .39$	$p < .0001$	-5598.53	.86	1.62
Profile-Varying Diagonal								
2	12029.47	12272.14	12065.98	$p = .40$	$p < .0001$	-5949.74	.75	46.3
Profile-Invariant Unrestricted								
2	10907.40	11538.34	11002.34	$p < .001$	Not replicated	-5824.70	.91	25.5
3	10896.61	11591.01	11001.09	$p = .69$	$p = .22$	-5262.31	.92	4.59
Profile-Varying Unrestricted								
2	11164.21	11496.48	11214.21	$p = .76$	$p < .0001$	-5492.11	.62	49.2

[a]Chosen as the optimal profile solution

Abbreviations: LPA = Latent Profile Analysis; AIC = Akaike Information Criterion; BIC = Bayesian Information Criterion; sBIC = sample-size adjusted Bayesian Information Criterion; VLMR-LRT = Vuong-Lo-Mendell-Rubin Likelihood Ratio Test; PBLRT = Parametric Bootstrap Likelihood Ratio Test

© 2025 Rowe J et al. *JAMA Network Open*.

eAppendix 3. Optimal Profile Solution Selection

All LPAs were initially performed with the default number of random starts (50, 5). The selected model was re-run with increased random starts (i.e., 100, 25; 500, 50) to ensure replication of the global maxima and protect against misidentification errors.

As seen in eTable1, within the Profile-Varying Diagonal structure, attempts to estimate models with more than two profiles failed to replicate the global maxima, indicating model instability. The two-profile solution within this structure exhibited suboptimal performance relative to the Profile-Invariant Diagonal covariance structure. Specifically, the entropy value was relatively low, and the VLMR-LRT was non-significant, suggesting that this model may not provide a better fit the one-profile solution.

In the Profile-Invariant Unrestricted model, the PBLRT for the two-profile solution was unreliable, raising concerns about overfitting. In turn, neither the VLMR-LRT nor the PBLRT results were significant for the 3-profile compared to the 2-profile solution. Estimating a four-profile solution resulted in non-convergence.

Attempts to estimate a three-profile solution within the Profile-Varying Unrestricted structure failed to converge, regardless of the number of random starts. The two-profile solution within this structure also demonstrated limited robustness, with a non-significant VLMR-LRT and notably low entropy, reflecting poor classification quality compared to the Profile-Invariant diagonal covariance structure.

As seen in eTable1, for the Profile-Invariant Diagonal covariance structure, the BIC and sBIC indices consistently decreased with the addition of more latent profiles. They did not converge on an optimal solution. This trend often indicates potential overfitting at a higher number of profiles, where the models begin to capture noise rather than underlying data patterns.[6,7] Although the AIC identified the six-profile solution as the best fit, the lack of convergence on optimal BIC and sBIC values warrants caution in interpreting these results. Additionally, the six-profile included a profile comprising only 6.14% of the sample, which raises concerns about over-extraction and instability at higher profile counts.[8]

The VLMR-LRT did not reach significance when comparing the three-profile solution to the two-profile solution. However, the VLMR-LRT indicated that the four-profile solution fit significantly better than the three-profile solution. Comparisons involving models with more than five profiles were non-significant, suggesting that additional profiles do not offer a meaningful improvement in model fit beyond four profiles. Entropy values were relatively consistent across models, exceeding .80 for the two-profile and four- through seven-profile models. This indicates that these models, except for the three-profile solution, demonstrated highly discriminative latent profiles.[9]

Taken together, the four-profile solution within the profile-invariant diagonal covariance structure is the most suitable model for this analysis. Within its covariance structure, the four-profile solution significantly improved fit over the three-profile model while maintaining a relatively high entropy value and reduced signs of overfitting. Moreover, this covariance structure estimate showed the most reliable results and contained no warnings regarding over-extraction or overfitting

© 2025 Rowe J et al. *JAMA Network Open*.

eTable 2. Descriptive Raw Score Statistics for the Clinical Variables in the Full Sample *(n = 309)*

Variable	*Statistic*
Clinical Course *M(SD)*	
Years of Morbidity	13.11(11.78)
Age at Onset	20.03(10.57)
No. of Episodes	2.99(3.60)
Comorbidities *n(%)*	
Anxiety Disorder	146(47.2)
Obsessive-Compulsive Disorder	12(3.9)
Post-Traumatic Stress Disorder	31(10.0)
Eating Disorder	15(4.9)
Symptom Domains *M(SD)*	
Negative Thoughts	6.12(2.98)
Detachment	8.19(3.54)
Neurovegetative Symptoms	7.92(3.07)
Sadness	6.66(1.92)

© 2025 Rowe J et al. *JAMA Network Open*.

eTable 3. Clinical Characteristics and Symptom Severity Differences Between Latent Profile Classes

Variable	Profile 1	Profile 2	Profile 3	Profile 4	Omnibus χ2 test	*p-value*
Course						
Years of Morbidity	11.58[9.28, 13.87][a]	10.31[7.99, 12.62][a,]	16.57[14.53, 25.28][a,b]	19.91[12.45, 20.69][b]	15.45	.001
Age at Onset	22.52[17.60, 27.43]	18.44[14.60, 22.28]	17.64[11.15, 24.13]	20.90[11.15, 24.13]	6.81	.08
No. of Episodes	2.76[2.09, 3.42]	2.29[1.66, 2.92]	3.76[2.80, 4.72]	4.63[1.85, 7.41]	8.03	.05
Comorbidities						
Anxiety	42.1%[30.3, 53.7]	56.6%[42.9, 70.3]	45.6%[31.9, 59.3][a]	41.2%[23.6, 58.8][a]	2.73	0.44
OCD	4.1%[0.1, 8.0]	2.4%[-1.0, 6.3][a]	5.0%[-2.8,12.8] [a]	5.8%[-2.0, 13.4][a]	1.04	0.79
PTSD	5.1%[-0.1, 11.0][a]	7.6%[-2.0, 13.6][a]	17.0%[5.2, 36.6][a]	20.9%[5.2, 28.8][a]	7.48	0.06
Eating	2.4%[-1.5, 6.3][a]	5.6%[-2.8, 12.8][a]	6.8[-1.0, 14.6][a]	7.0%[-2.8, 16.8][a]	2.08	0.55
Symptom Severity						
Neg. Thoughts	4.39[4.00, 4.78]	4.56[4.11, 5.01]	4.75[4.04, 5.46]	5.20[4.49, 5.91]	4.18	.24
Detachment	9.21[8.68, 9.73][a]	9.98[9.45, 10.51][a,b]	9.51[8.51, 10.51][a,b]	10.72[9.74, 11.70][b]	29.03	.03
Neurovegetative	7.52[6.93, 8.11]	7.99[7.25, 8.73]	8.25[7.39, 9.11]	8.35[7.23, 9.47]	2.79	.43
Low Mood	5.98[5.57, 6.39][a]	7.04[6.63, 7.45][b]	6.96[6.47, 7.45][b]	7.19[6.54, 7.84][b]	15.96	.001
Remission Status						
Week 8	29.4%[15.3, 42.7]	30.8%[17.1, 44.5]	34.3%[10.8, 57.8]	30.3%[12.7, 47.9]	0.11	.99
Week 16	64.0%[46.3, 81.6]$_{a}$	48.9%[9.7, 88.1]$_{a,b}$	90.9%[63.4, 118.0]$_{c}$	21.5%[17.6, 23.5]$_{b}$	16.63	.001

Note. Data summarized as mean (continuous variables) or probability of class membership (categorical variables) and [95% CI]. Superscript letters indicate significant pairwise differences between profiles based on Wald χ^2 parameter constraint tests ($p < .008$, Bonferroni-corrected). Profiles that do not share a superscript are significantly different from one another ($p < .008$); profiles that share the same superscript do not significantly differ.

© 2025 Rowe J et al. *JAMA Network Open*.

eReferences.

[1] Sämann PG, Iglesias JE, Gutman B, et al. FreeSurfer-based segmentation of hippocampal subfields: A review of methods and applications, with a novel quality control procedure for ENIGMA studies and other collaborative efforts. *Human Brain Mapping*. 2022;43(1):207-233. doi:10.1002/hbm.25326

[2] Nordenskjöld R, Malmberg F, Larsson EM, et al. Intracranial volume normalization methods: Considerations when investigating gender differences in regional brain volume. *Psychiatry Research: Neuroimaging*. 2015;231(3):227-235. doi:10.1016/j.pscychresns.2014.11.011

[3] Churchill, N. W., Spring, R., Afshin-Pour, B., Dong, F., & Strother, S. C. (2015). An automated, adaptive framework for optimizing preprocessing pipelines in task-based functional MRI. PloS one, 10(7), e0131520.

[4] Churchill, N. W., Raamana, P., Spring, R., & Strother, S. C. (2017). Optimizing fMRI preprocessing pipelines for block-design tasks as a function of age. *NeuroImage*, *154*, 240-254.

[5] Raamana, Pradeep Reddy, Churchill, Nathan W., & Strother, Stephen C.. Optimization of Preprocessing Pipelines for NeuroImaging (OPPNI) for fMRI preprocessing (Version v0.7.3.1_06JUL2017). Zenodo. http://doi.org/10.5281/zenodo.3662956

[6] Nylund, K. L., Asparouhov, T., & Muthén, B. O. (2007). Deciding on the Number of Classes in Latent Class Analysis and Growth Mixture Modeling: A Monte Carlo Simulation Study. *Structural Equation Modeling: A Multidisciplinary Journal*, *14*(4), 535–569. https://doi.org/10.1080/10705510701575396

[7] Nylund-Gibson, K., Grimm, R. P., & Masyn, K. E. (2019). Prediction from Latent Classes: A Demonstration of Different Approaches to Include Distal Outcomes in Mixture Models. *Structural Equation Modeling: A Multidisciplinary Journal*, *26*(6), 967–985. https://doi.org/10.1080/10705511.2019.1590146

[8] Nylund-Gibson, K., & Choi, A. Y. (2018). Ten frequently asked questions about latent class analysis. *Translational Issues in Psychological Science*, *4*(4), 440–461. https://doi.org/10.1037/tps0000176

[9] Weller, B.E., Bowen, N. K. & Faubert, S.J. (2020). Latent Class Analysis: A Guide to Best Practice. *Journal of Black Psychology*, *46*(4), 287-311. https://doi.org/10.1177/0095798420930932

© 2025 Rowe J et al. *JAMA Network Open*.
