# Supplementary material for: Latent Profile Analysis of Childhood Maltreatment and Neural Markers in Depression
Source: JAMA Netw Open. 2025 Aug 4;8(8):e2525147. doi: 10.1001/jamanetworkopen.2025.25147 (PMC12322798; doi:10.1001/jamanetworkopen.2025.25147)
Supplement: Supplement 2. — Data Sharing Statement [file jamanetwopen-e2525147-s002.pdf]

Data Sharing Statement

Rowe. Latent Profile Analysis of Childhood Maltreatment and Neural Markers in Depression. *JAMA Netw Open*. Published August 04, 2025. doi:10.1001/jamanetworkopen.2025.25147

Data
Data available: Yes
Data types: Deidentified participant data, Data dictionary
How to access data: Deidentified participant data and the data dictionary for CAN-BIND-1 are available through braincode.ca (see below for data access specifications). While CAN-BIND-4 data is not currently accessible, efforts are underway in collaboration with Brain-CODE and the Ontario Brain Institute to enable its availability in the future.
When available: beginning date: 01-13-2023

Supporting Documents
Document types: None

Additional Information
Who can access the data: The data will be made available to two types of users: OBI-Funded Investigators: These individuals will have accounts set up by Indoc, granting them access to data they have generated and from studies in which they are listed as collaborators. Access to controlled data that they did not generate or collaborate on requires the completion of a Data Access Request and approval by the Data Access Committee (DAC). External Users: External researchers can request access by contacting help@braincode.ca. This process requires the applicant to provide verified contact information and association. Access to any non-public data also requires the completion of a Data Access Request and approval by the DAC.
Types of analyses: The data will be made available for analyses that adhere to the Ontario Brain Institute's (OBI) Governance Policies, all Applicable Laws and Guidelines (including PHIPA), and demonstrate scientific merit. The Data Access Committee (DAC) will review requests to ensure the appropriateness of the data for the intended use, including an assessment of the researcher's affiliations and hypothesis. Data access will only be approved for purposes aligned with these criteria.
Mechanisms of data availability: Data will be made available after the approval of a proposal and upon signing a Data Use Agreement. This agreement includes applicable confidentiality requirements and provisions prohibiting any attempts to re-identify the data.
